# Regionally selective requirement for D_1_/D_5_ dopaminergic neurotransmission in the medial prefrontal cortex in object-in-place associative recognition memory

**DOI:** 10.1101/lm.036921.114

**Published:** 2015-02

**Authors:** Giorgia Savalli, Zafar I. Bashir, E. Clea Warburton

**Affiliations:** 1 Departments of Neurophysiology and Neuropharmacology, Medical University of Vienna, 1090 Vienna, Austria; 2 Department of Physiology and Pharmacology, School of Medical Sciences, University of Bristol, Bristol BS8 1TD, United Kingdom

## Abstract

Object-in-place (OiP) memory is critical for remembering the location in which an object was last encountered and depends conjointly on the medial prefrontal cortex, perirhinal cortex, and hippocampus. Here we examined the role of dopamine D_1_/D_5_ receptor neurotransmission within these brain regions for OiP memory. Bilateral infusion of D_1_/D_5_ receptor antagonists SCH23390 or SKF83566 into the medial prefrontal cortex, prior to memory acquisition, impaired OiP performance following a 5 min or 1 h delay. Retrieval was unaffected. Intraperirhinal or intrahippocampal infusions of SCH23390 had no effect. These results reveal a selective role for D_1_/D_5_ receptors in the mPFC during OiP memory encoding.

Object-in-place (OiP) associative recognition memory involves the formation of an association between an object and the location in which it was last encountered (Gaffan and Parker 1996; Dix and Aggleton 1999) and is therefore a key component of event memory (Mecklinger and Meinshausen 1998). The medial prefrontal cortex (mPFC), perirhinal cortex (PRH), and hippocampus (HPC), comprise an associative recognition memory neural circuit (Gaffan 1994; Browning et al. 2005; Barker et al. 2007; Bachevalier and Nemanic 2008; Barker and Warburton 2013; Lee and Park 2013). However, the neural mechanisms, which underlie the formation of OiP memory, are currently underexplored. The mPFC, PRH, and HPC all receive prominent dopaminergic innervation (Berger et al. 1974; Scatton et al. 1980; Swanson 1982; Sobel and Corbett 1984; Fallon and Laughlin 1995; DiChiara 2002) and exposure to novel stimuli and novel environments increases midbrain dopaminergic cell body firing (Feenstra et al. 1995; Beaufour et al. 2001; De Leonibus et al. 2006). Chao et al. (2013) recently reported that a unilateral forebrain dopamine lesion combined with a unilateral mPFC lesion significantly impaired OiP memory. Thus dopamine is a strong candidate for driving novelty processing, critical during recognition memory. Dopamine acts through different receptor subtypes (D_1_–D_5_) located within the mPFC, HPC, and PRH, and intra-PRH infusion of the D_1_/D_5_ receptor antagonist SCH23390 impaired object recognition after 24 h but not 90 min (Balderas et al. 2013). Thus here we examined the importance of D_1_/D_5_ receptor neurotransmission, selectively within the mPFC, PRH, and HPC, during recognition memory encoding or retrieval.

Rats were implanted with bilateral cannulae aimed at the mPFC, HPC, or PRH to allow direct intracerebral administration of the D_1_/D_5_ receptor antagonists SCH23390 or SKF83566. All animal procedures were performed in accordance with the United Kingdom Animals Scientific Procedures Act (1986) and associated guidelines. Details of the surgery, infusion procedures, behavioral testing, and histology have been published previously (Barker and Warburton 2008). Briefly, male Dark Agouti rats (230–250 g; Harlan, UK) housed under a 12-h/12-h light/dark cycle (light phase 18:00–6:00 h), were anesthetized with isoflurane (induction 4%, maintenance 2%–3%) and bilateral cannulae were surgically implanted at these coordinates relative to bregma: PRH: anterior–posterior (AP) −5.6 mm; mediolateral (ML) ±4.47 mm; dorsoventral (DV) −6.7 mm (relative to the skull) at an angle of 20° to the vertical; mPFC: AP +3.20 mm; ML ±0.75 mm; DV −3.5 mm; HPC: AP −4.8 mm; ML ±2.6 mm; DV −3.0 mm. After recovery and habituation all rats were tested in the following tasks: object-in-place (OiP), novel object recognition (NOR) and object location (OL), within an arena (50 × 90 × 100 cm). All tasks involved a sample and test phase, separated by a 5 min or 1 h delay. The objects presented were constructed from “Duplo” (Lego, UK Ltd.) and placed 15 cm from the arena walls. Exploratory behavior was defined as the animal directing its nose toward the object at a distance of <2 cm.

To assess OiP memory, subjects were presented with four different objects (Fig. 1Ai) in the sample phase (5 min). At test (3 min), two objects exchanged positions, and the time subject spent exploring the objects that had changed position was compared with the time spent exploring the objects in the same position. Object and position were counterbalanced across rats. OiP memory is intact when the subject spends more time exploring the moved compared with the stationary objects. To assess NOR memory, duplicate objects were placed in the arena in the sample phase (Fig. 1Aii). At test a copy of the sample phase object and a novel object were presented and exploration of the objects compared. To assess OL memory, duplicate objects were placed in the arena (Fig. 1Aiii). At test, one object was placed in the same position as in the sample phase while a second was placed in the corner adjacent to its original position.

**Figure 1.**
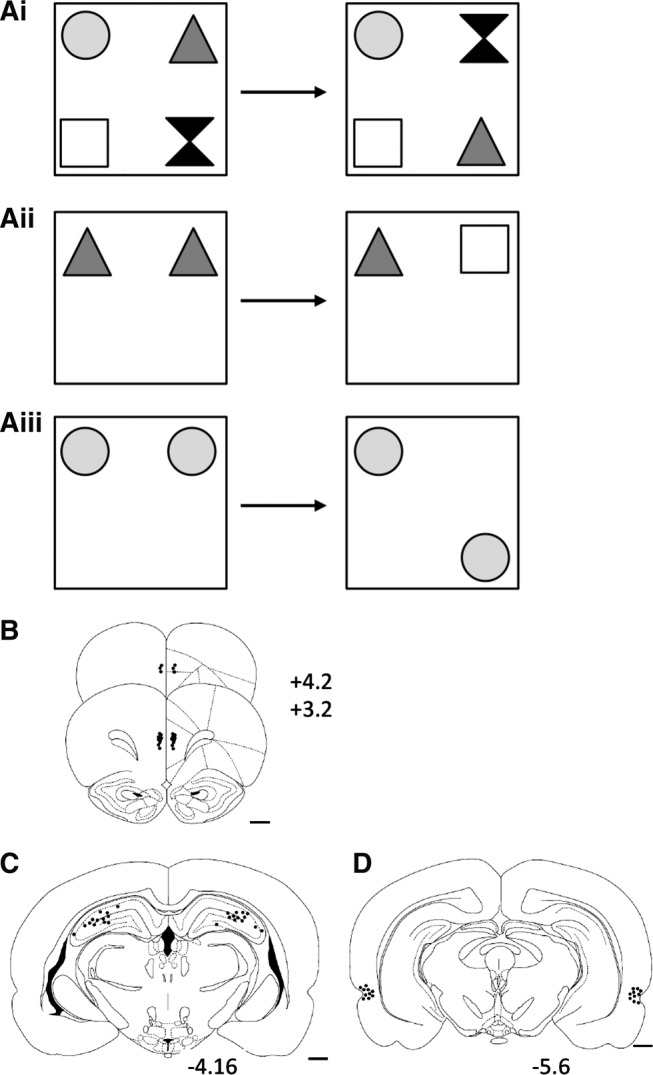
Diagram of the three object recognition memory tasks and of the histology of the individual infusion sites. (*Ai*) Object-in-place (OiP) task. (*Aii*) Novel object recognition (NOR) task. (*Aiii*) Object location (OL) task. (*B*) Bilateral mPFC group. (*C*) Bilateral HPC group. (*D*) Bilateral PRH group. The numbers correspond to the approximate position from bregma (Paxinos and Watson 1998). Scale bar, 1 mm.

Each experiment consisted of two sessions separated by a minimum of 48 h. Vehicle or drug was infused in a cross-over design and each animal retested using different objects.

Infusions were given 15 min before the sample phase (to assess effects on acquisition) or 15 min before the test phase (to assess effects on retrieval). SCH23390 hydrochloride (Tocris Bioscience) dissolved in sterile 0.9% saline solution (Aquapharm) was administered at a concentration of 5 mM per hemisphere based on previous studies (Seamans et al. 1998; Romanides et al. 1999; Baldwin et al. 2002; Izquierdo et al. 2007; Winter et al. 2009). SKF83566 hydrobromide (Tocris Bioscience) dissolved in sterile saline (0.9%) was administered at a concentration of 200 µM per hemisphere. No previous studies have administered SKF83566 into selective brain regions in vivo, so the dose used was based on in vitro electrophysiological studies showing SKF83566 to have effects on D_1_/D_5_ receptors at doses between 2 and 10 μM (Yamamoto et al. 2007; Stramiello and Wagner 2008). Drugs were infused over 2 min into the mPFC and PRH at a rate of 0.5 μL/min and into the HPC at a rate of 0.25 μL/min. The infusion cannulae remained in place for an additional 5 min.

On completion of the experiments each rat was anesthetized and perfused transcardially. Coronal brain sections (40 µm) were stained with cresyl-violet to verify the cannulae locations. Rats in the mPFC group all had cannulae tips in the ventral portion of the prelimbic or dorsal portion of the infralimbic region of the prefrontal cortex (Fig. 1B). All rats in the HPC and PRH group had the tip of the cannulae within the intended region (Fig. 1C,D).

Presample administration of SCH23390 into the mPFC profoundly impaired OiP memory [main effect of treatment: (*F*_(1,16)_ = 18.27, *P* < 0.01), Fig. 2Ai] irrespective of the retention delay [treatment × delay: (*F*_(1,16)_ = 1.63, ns). A within subjects *t*-test (two-tailed) confirmed that, at both delays, the vehicle group showed a significant preference for the moved objects (5 min *t*_(7)_ = 4.41, *P* < 0.01; 1 h *t*_(9)_ = 2.91, *P* < 0.05) while the SCH23390 group did not (5 min *t*_(7)_ = −0.39, ns; 1 h *t*_(9)_ = −0.54, ns). SCH23390 was without effect on the total amount of exploration in the sample (5 min *F*_(1,7)_ = 1.67, ns; 1 h *F*_(1,9)_ = 0.28, ns) or test phases (5 min *F*_(1,7)_ = 0.27, ns; 1 h *F*_(1,9)_ = 0.71, ns). Intra-mPFC administration of SKF83566 prior to the sample phase impaired OiP memory [Fig. 2Aii; main effect of treatment (*F*_(1,20)_ = 22.72, *P* < 0.001)] irrespective of the delay [treatment × delay interaction (*F*_(1,20)_ = 0.403, ns)]. The vehicle-treated group showed a significant preference for the moved over the stationary objects (5 min *t*_(11)_ = 9.56, *P* < 0.001; 1 h *t*_(9)_ = 5.29, *P* < 0.01) while the SKF83566-treated group did not (5 min *t*_(11)_ = 1.32, ns; 1 h *t*_(9)_ = −0.60, ns). SKF83566 was without effect on the total amount of exploration in the sample (5 min *F*_(1,11)_ = 1.34, ns; 1 h *F*_(1,9)_ = 0.22, ns) or test phases (5 min *F*_(1,11)_ = 0.06, *P* > 0.05; 1 h *F*_(1,9)_ = 1.65, *P* > 0.05).

**Figure 2.**
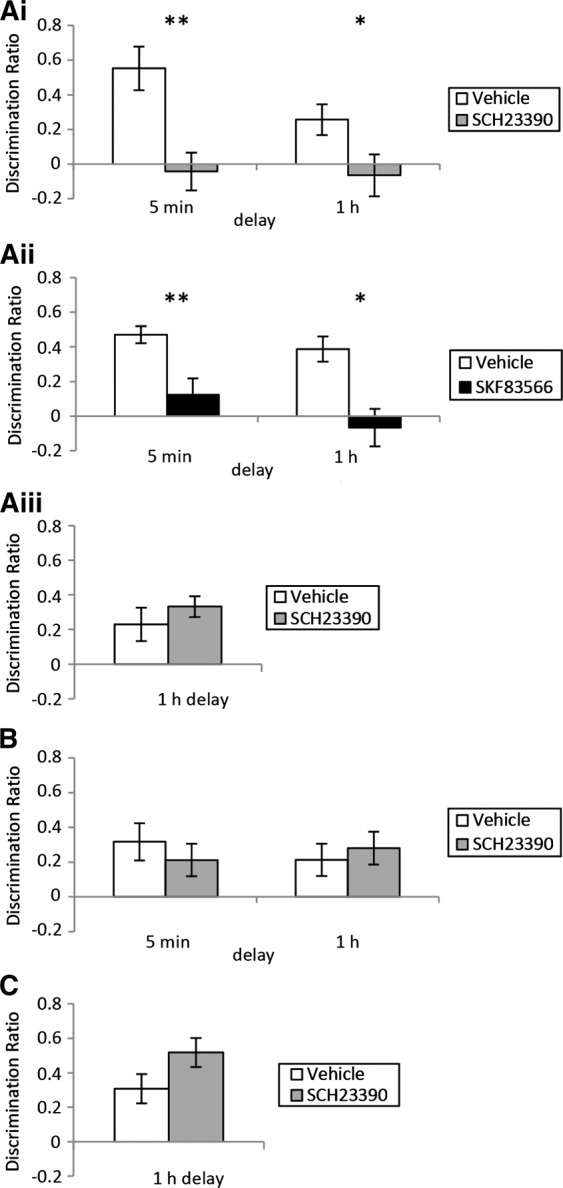
Performance of mPFC (*A*), HPC (*B*), and PRH (*C*) groups in the object-in-place (OiP) task. Discrimination between the objects was calculated using a discrimination ratio, which is calculated as follows: the difference in time spent by each animal exploring objects that changed position compared with the objects that remained in the same position, divided by the total time spent exploring all objects. (*Ai*) Infusion of SCH23390 into the mPFC before the sample phase significantly impaired performance in the OiP task following a 5 min (*n* = 8) and a 1 h (*n* = 10) delay. (*Aii*) Infusion of SKF83566 into the mPFC before the sample phase significantly impaired performance in the OiP task following a 5 min (*n* = 12) and a 1 h (*n* = 10) delay. (*Aiii*) Infusion of SCH23390 into the mPFC before the test phase had no effect on OiP performance after a 1 h delay (*n* = 9). (*B*) Infusion of SCH23390 into the HPC before the sample phase had no effect on OiP performance after a 5 min (*n* = 6) or a 1 h (*n* = 10) delay. (*C*) Infusion of SCH23390 into the PRH before the sample phase had no effect on OiP performance after a 1 h delay (*n* = 10). Illustrated for each group is the mean (± SEM) discrimination ratio. (*) *P* < 0.05; and (**) *P* < 0.01 difference between groups.

The impairment produced by antagonism of D_1_/D_5_ receptors in the mPFC could reflect an effect on retrieval as well as acquisition as the drug is likely to be present during both sample and test. To examine potential effects on retrieval, SCH23390 or vehicle was infused into the mPFC 15 min before the test phase, which occurred 1 h following the sample phase. No significant impairment in memory performance was found [*F*_(1,8)_ = 1.51, ns; Fig. 2Aiii] and both the vehicle- and SCH23390-treated groups discriminated between the moved and stationary objects (vehicle *t*_(8)_ = 2.36, *P* < 0.05; SCH23390 *t*_(8)_ = 5.47, *P* < 0.001). There were no significant differences in the total amount of exploration completed in the sample (*F*_(1,8)_ = 1.21, ns) or test phases (*F*_(1,8)_ = 1.13, ns).

We next examined the requirement for D_1_/D_5_ receptors in the HPC or PRH for the acquisition of OiP. Intra-HPC infusion of SCH23390 prior to the sample phase had no effect on performance [*F*_(1,21)_ = 0.042, ns; Fig. 2B] at either delay [treatment × delay *F*_(1,21)_ = 0.86, ns]. Both the vehicle- and the SCH23390-treated groups showed a significant preference for the moved over the stationary objects (5 min vehicle: *t*_(11)_ = 2.96, *P* < 0.05; SCH23390: *t*_(11)_ = 2.27, *P* < 0.05; 1 h vehicle: *t*_(10)_ = 2.30, *P* < 0.05; SCH23390: *t*_(10)_ = 2.98, *P* < 0.05). Intra-PRH infusion of SCH23390, before the sample phase also had no effect on OiP performance following a 1 h delay [*F*_(1,9)_ = 4.21, ns; Fig. 2C] and both the vehicle- (*t*_(9)_ = 3.59, *P* < 0.01) and the SCH23390- (*t*_(9)_ = 6.21, *P* < 0.001) treated animals showed significant discrimination.

To examine whether the OiP impairment following blockade of mPFC D_1_/D_5_ receptors could be explained by changes in levels of alertness or arousal we examined the effects of presample intra-mPFC administration of SCH23390 on NOR, a task which uses using the same apparatus, stimulus types, and delay but does not depend on the mPFC (Hannesson et al. 2004; Barker et al. 2007). No significant difference in performance between the groups was observed following a 1 h delay [*F*_(1,9)_ = 1.78, *n.s*; Fig. 3A] further both groups preferentially explored the novel over the familiar object (vehicle: *t*_(9)_ = 7.55, *P* < 0.001; SCH23390: *t*_(9)_ = 4.27, *P* < 0.01). Interestingly there was also no effect of presample intra-PRH infusion of SCH23390 on NOR performance [*F*_(1,6)_ = 0.04, ns; Fig. 3B] and both groups preferentially explored the novel compared with the familiar object (vehicle *t*_(6)_ = 4.3, *P* < 0.01; SCH23390 *t*_(6)_ = 5.0, *P* < 0.01).

**Figure 3.**
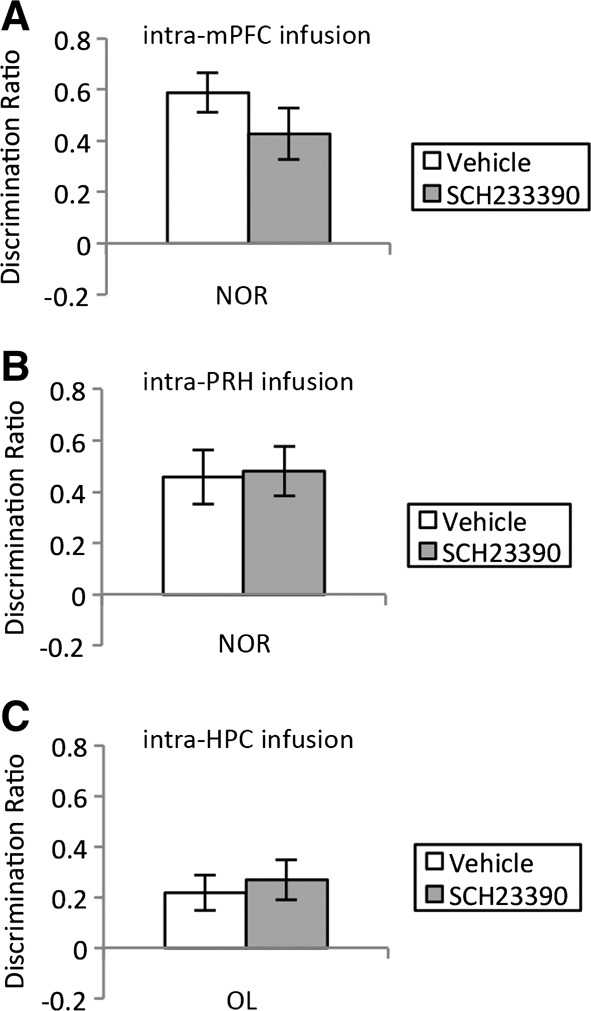
(*A*) Infusion of SCH23390 into the mPFC before the sample phase had no effect on NOR performance after 1 h delay (*n* = 10). (*B*) Infusion of SCH23390 into the PRH before the sample phase had no effect on NOR performance after 1 h delay (*n* = 7). (*C*) Infusion of SCH23390 into the HPC before the sample phase had no effect on OL performance after 1 h delay (*n* = 10). Illustrated for each group is the mean (±SEM) discrimination ratio.

Finally we examined the effects of hippocampal D_1_/D_5_ receptor blockade on the acquisition of OL memory, a task dependent on the HPC (Barker and Warburton 2011). Infusion of SCH23390 into HPC had no effect on OL after 1 h delay [*F*_(1,9)_ = 0.214, ns; Fig. 3C] and both the vehicle- and SCH23390-treated groups preferentially explored the object in the new location (vehicle: *t*_(9)_ = 3.10, *P* < 0.05; SCH23390: *t*_(9)_ = 3.39, *P* < 0.01).

Presample infusion of D_1_/D_5_ receptor antagonists into the mPFC, but not PRH or HPC impaired the acquisition of short (5 min) and longer-term (1 h) OiP memory. D_1_/D_5_ receptor blockade in the PRH and HPC had no effect on NOR or OL memory, respectively, following a 1 h delay (hence shorter delays were not examined). Together these results demonstrate that dopaminergic neurotransmission via D_1_-like receptors in the mPFC is selectively required for the acquisition of OiP associative recognition memory, and these receptors appear not to be involved in the formation of other types of recognition memory.

Blockade of D_1_/D_5_ receptors in the mPFC, PRH or HPC was without effect on exploration in any of the recognition tasks used. Furthermore, D_1_/D_5_ blockade in either the mPFC or PRH had no effect on NOR, thus the memory impairments cannot be attributed to a general impairment of arousal or changes in locomotor activity. A number of studies demonstrate permanent or temporary lesions of the mPFC or specific catecholaminergic depletion within the mPFC does not affect NOR (Mitchell and Laiacona 1998; Hannesson et al. 2004; Barker et al. 2007; Nelson et al. 2011; Cross et al. 2012) suggesting that single item recognition does not depend on the mPFC. In contrast one study has shown that SCH23390 administration into the prelimbic cortex dose-dependently impaired NOR following a 5 min delay (Clausen et al. 2011) and at present there is no clear reason for the discrepancy between this study and the present results, although the present study used a higher drug concentration (5 mM compared with 0.05–0.5 nM) and different strains of rats were used in the two studies.

OiP memory requires a functional interaction between the HPC and mPFC (Barker and Warburton 2011, 2013) and dopamine has been shown to modulate cognitive function through regulation of synaptic transmission and plasticity in both regions (Seamans et al. 1998; Gurden et al. 2000; Xu and Yao 2010). That blockade of D_1_/D_5_-like receptors in the HPC had no effect on OiP or OL was surprising as D_1_-like receptors are the primary dopaminergic subtype in HPC (Köhler et al. 1991; Laurier et al. 1994) and have been shown to be critical for the modulation of hippocampal synaptic plasticity (Lemon and Manahan-Vaughan 2006). Further D_1_/D_5_ receptor blockade in the HPC impaired spatial learning (O'Carroll et al. 2006; Ortiz et al. 2010). It might be that hippocampal D_1_/D_5_ receptors are required in tasks that require different response strategies, i.e., the OL task is based on an animal's spontaneous preference for novelty, while the water maze involves extensive training. Another issue is the length of retention delay. Thus SCH23390 impaired water maze performance after a 6 h delay, but not after 20 min (O'Carrol et al. 2006) and intra-PRH administration of SCH23390 impaired NOR at 24 h, but not at 90 min (the latter result consistent with the present findings) (Nagai et al. 2007; Balderas et al. 2013). These studies suggest a role for D_1_/D_5_ receptors in the PRH or HPC for memory retention over relatively long delays. Importantly however, our results reveal that at a 1 h delay there is a critical regional difference between the mPFC, PRH, and HPC in the requirement for D1/D5 receptor neurotransmission for OiP memory.

One explanation for the regional selectivity can be ascribed to a potential specific function of the mPFC, compared with the PRH or HPC, in OiP memory. Previous studies suggest that the PRH is critical for processing object identity information or the relative familiarity of the stimuli (Barker and Warburton 2008), while the mPFC integrates object and location information required for OiP (Barker et al. 2007). Hence one may hypothesize that D_1_/D_5_ receptor activation in the mPFC is crucial for the plasticity processes, which underlie the formation of the object–place associations and guide behavior in the OiP task. In contrast, the information processing in the PRH and HPC occurs independent of dopamine neuromodulation via D_1_/D_5_ receptors.

Both the D_1_/D_5_ antagonists used in this study show affinity for serotonin receptors and it may be argued that the deficits observed in this study could reflect a disruption of serotoninergic neurotransmission on learning and memory (Molodtsova 2008). SCH23390 is a 5-HT_2C_ receptor agonist (Millan et al. 2001) while SKF83566 is a 5-HT_2C_ receptor antagonist (Ohlstein and Berkowitz 1985). However, previous studies have shown that the systemic administration of ketanserin, a 5-HT_2C_ receptor antagonist, improved memory performance (Meneses and Hong 1997; Ruotsalainen et al. 1997) thus it is unlikely that the impairment observed in the present study is due to an action at 5-HT_2C_ receptors. The present study did not consider the role D_2_ receptors, although systemic administration of the D_2_ receptor antagonist quinpirole has been shown to have no effect on NOR (de Lima et al. 2011) suggesting this dopamine receptor subtype may be less critical in recognition, however, this question should be addressed directly.

D_1_/D_5_ receptor antagonism in the mPFC, but not in the PRH or HPC, disrupts OiP memory performance. OiP performance is disrupted in schizophrenic patients (Wood et al. 2002), in animal models of schizophrenia (Howland et al. 2012), and drug abuse (Reichel et al. 2014), all of which are linked with disturbances in dopaminergic neurotransmission. The regionally selective requirement for dopamine neurotransmission mediated by D_1_/D_5_ receptors suggests different neural mechanisms underlie the formation of OiP recognition memory within the PRH–HPC–mPFC circuit. In addition, these results highlight the utility of the OiP task to explore links between associative memory processes and cognitive function associated with dopamine neurotransmission.
